# Driving after alcohol consumption among residents of Northeastern Brazil: National Health Survey 2019

**DOI:** 10.1590/S2237-96222024v33e2024455.en

**Published:** 2024-10-14

**Authors:** Renata da Silva Gomes, Amanda Cristina de Souza Andrade, Vanessa Moraes Bezerra

**Affiliations:** 1 Universidade Federal da Bahia, Programa de Pós-Graduação em Saúde Coletiva, Vitória da Conquista, BA, Brazil; 2 Universidade Federal de Mato Grosso, Instituto de Saúde Coletiva, Cuiabá, MT, Brazil

**Keywords:** Accidentes de Tránsito, Estudios Transversales, Consumo de Bebidas Alcohólicas, Conductas de Riesgo para la Salud, Encuestas Epidemiológicas, Traffic Accidents, Cross-sectional Studies, Consumption of Alcoholic Beverages, Health Risk Behaviors, Epidemiological Surveys

## Abstract

**Objective:**

To estimate the prevalence of factors associated with drinking and driving in Northeastern Brazil.

**Methods:**

This was a cross-sectional study conducted with participants from the 2019 National Health Survey, aged ≥ 18 years; the analysis of the association between sociodemographic variables and the outcome, stratified by sex, was performed using Poisson regression.

**Results:**

The prevalence of drinking and driving was 21.0% ( 95%CI 19.9;23.2), with 24.6% ( 95%CI 22.7;26.5), in males and 10.1 % ( 95%CI 7.9;12.7), in females (p-value < 0.001); among men, younger age groups (PR = 1.70 – 95%CI 1.29;2.24), higher household income (PR = 1.74 – 95%CI 1.33;2.28), rural residence (PR = 1.48 – 95%CI 1.26;1.74) and motorcycle riding (PR = 1.29 – 95%CI 1.05;1.58) were associated with the event, while no association was observed among women.

**Conclusion:**

Prevalence of drinking and driving was high in the Northeast region, especially among the male population; preventive measures targeting this group and intensified enforcement are necessary.

## Study contributions

**Main results:**

The prevalence of drinking and driving was high, significantly greater in males when compared to females; positive associations between sociodemographic variables and the event were observed only in the male population.

**Implications for services:**

Intersectoral actions, involving health services and regulatory bodies, are essential to reduce the habit of drinking and driving, especially among males, in addition to promoting a safer road environment in the Northeast region.

**Perspectives:**

It is necessary to strengthen enforcement measures for alcohol consumption and driving, especially in rural areas and among motorcycle riders, in addition to implementing traffic education programs and awareness campaigns.

## RESUMEN

**Objetivo:**

Estimar la prevalencia y analizar factores asociados al consumo de alcohol y conducir en el Nordeste de Brasil.

**Métodos:**

Estudio transversal con participantes de la Encuesta Nacional de Salud 2019, ≥ 18 años; se analizó la asociación entre variables sociodemográficas y el resultado, estratificadas por sexo, mediante regresión de Poisson.

**Resultados:**

La prevalencia de beber y conducir fue del 21,0% (IC95% 19,9;23,2), 24,6% (IC95% 22,7;26,5) en hombres y 10,1% (IC95% 7,9;12,7) en mujeres (p-valor < 0,001); en hombres, franja etaria más joven (RP = 1,70 – IC95%1,29;2,24), mayor ingreso familiar (RP = 1,74 –IC95% 1,33;2,28), en áreas rurales (RP = 1,48 – IC95% 1,26;1,74) y conducir motocicleta (RP = 1,29 – IC95% 1,05;1,58) se asociaron al evento; en las mujeres no se observaron asociaciones.

**Conclusión:**

La prevalencia de beber y conducir fue alta en el Nordeste específicamente entre hombres. Son necesarias medidas preventivas para esta población e intensificación de la supervisión.

## INTRODUCTION

Alcohol is a licit drug, widely used by both men and women across various cultures, associated with festivities, religious ceremonies and celebrations. The consumption of high amount of alcohol can cause damage to personal health and to society as a whole.^1,2^

Driving after consuming alcohol is one of the leading causes of traffic accidents in the world.^2^ In high-income countries, approximately 20% of fatally injured drivers had blood alcohol concentration, while in some low- and middle-income countries, this percentage was three times higher.^2^

Even in small amounts, alcohol induces psychomotor changes, such as the deterioration of visual and motor functions, in addition to reducing the capacity for discernment, posing substantial risks to the driver, passengers, and pedestrians.^3,4^ Regarding the risk factors associated with drinking and driving, male gender, younger age groups, and higher income levels stand out.^4,5^

In Brazil, traffic accidents were the leading cause of hospitalizations related to alcohol consumption in 2021 – 22.6% of the total – and the second most prevalent cause of mortality associated with the use of this substance – 15.8 % of total.^6^ The highest mortality rates from traffic accidents in the country were found in the North, Northeast and Midwest macro-regions, primarily affecting men, younger individuals and those with higher income.

There was a 43% reduction in mortality due to traffic accidents in Brazil between 1990 and 2019.^6^ Despite this significant progress, the goal of reducing deaths from traffic accidents by 50% in Brazil by 2030 may not be achieved .^7^

In addition to high traffic mortality, especially among motorcyclists, Northeastern Brazil faces profound inequalities, especially related to social and economic conditions. The region is known for having lower levels of education and income, and poorer health conditions. These factors are not only associated with high alcohol consumption but also pose a risk for drinking and driving.^8,9^

High morbidity and mortality from traffic accidents due to alcohol consumption and their consequences extend beyond hospital expenses and health care costs. ^4,10^ This issue poses a public health challenge worldwide, and Northeastern Brazil is not immune to it.

Despite the evident relevance and potential negative impacts on individual health and society, there is a significant gap in the scientific literature investigating this phenomenon in the Northeast region. The scarcity of studies on alcohol consumption in the region limits the understanding of consumption patterns, the risk factors involved and the consequences for public health.

This study aimed to evaluate the prevalence and sociodemographic factors associated with drinking and driving among residents of Northeastern Brazil, stratified by sex.

## METHODS

This was a cross-sectional study using data from the National Health Survey (*Pesquisa Nacional de Saúde* - PNS), 2019 edition,^11^ on adults living in the Northeast region of Brazil.

PNS 2019 data collection took place between August 2019 and March 2020. The survey adopted a three-stage cluster sampling design: census tracts; households; and an individual aged 15 years and older, residing in the household, randomly selected.^11,12^

**Figure 1:**
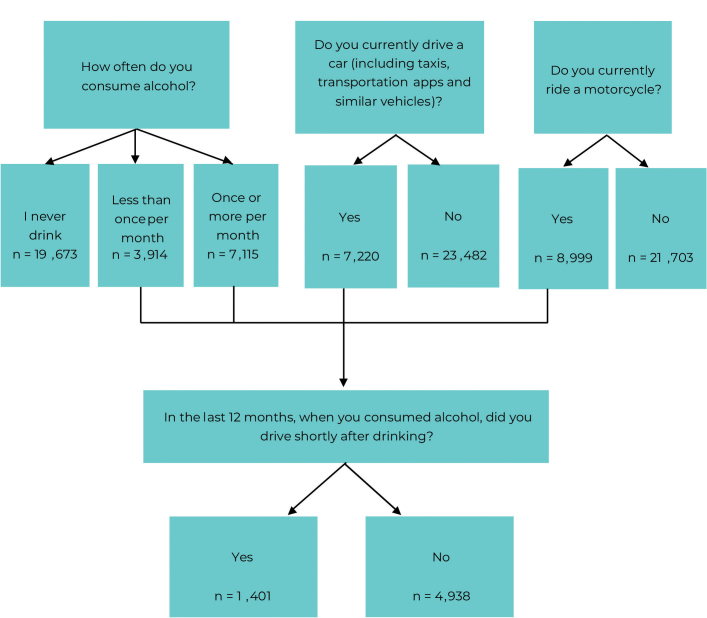
Process of identifying Individuals aged 18 years and older who reported drinking and driving, National Health Survey, Northeastern Brazil, 2019

In total, the PNS 2019 included 91,683 respondents aged 18 years and older, of whom 31,800 were residents of the Northeast region.^12^ In this study sample, those aged 18 years and older who answered questions about alcohol consumption, and those who drove cars or rode motorcycles were taken into consideration, totaling 6,339 individuals (Figure 1).

The dependent variable of the study was “drinking and driving” as a habit. This indicator was constructed based on the following PNS question: *On any of these days when you consumed alcohol, did you drive shortly after drinking?* The question had two response options, “yes” and “no”, asked only to individuals who reported consuming alcohol and those who drove a car or rode a motorcycle. Drivers were identified by the PNS using the following questions: *Do* *you currently drive a car (including taxis, transportation apps and similar vehicles)?* and *Do you currently ride a motorcycle?*

The analysis was stratified by the variable “sex” (male; female). Drinking and driving was analyzed according to sociodemographic characteristics:

age group (in years: 18 to 34; 35 to 59; 60 or more);
race/skin color (White and Black [Black and mixed-race]); Asian and Indigenous were excluded;
marital status (without a partner; with a partner);
education level (no education; elementary [incomplete/complete]; high school [incomplete/complete]; higher education [incomplete/complete]);
per capita household income (in minimum wages: ≤ ½; ½ to 1; >1 to 2; > 2);
place of residence (urban area; rural area);
drive a car (yes; no); and
ride a motorcycle (yes; no).

For data analysis, the Stata /SE software, version 15, and the survey module, which considers the complex sample design and sample weights. The prevalence of the event was calculated according to sociodemographic characteristics. Bivariate and adjusted analyses were performed, with prevalence ratios (PR) estimates and respective 95% confidence intervals (95%CI), using Poisson regression with robust variance. Multiple analysis was performed by means of the backward selection method, with all sociodemographic variables included; one variable at a time was eliminated from the model. Only variables with a p-value ≤ 0.05 were kept in the final model.

The 2019 National Health Survey project was approved by the National Research Ethics Committee of the National Health Council under opinion No. 3,529,376 of 23/08/2019; certificate of submission for ethical appraisal: 11713319.7.0000.0008.

## RESULTS

**Figure 2:**
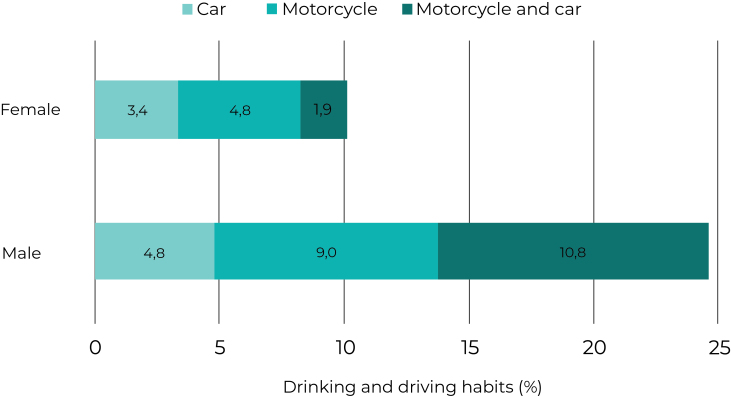
Prevalence of drinking and driving, according to the type of vehicle, in the population aged 18 years and older, stratified by sex, National Health Survey, Northeastern Brazil, 2019

Of the total respondents (n = 6,339 individuals), 79.0% were male and 21.0% were female; the prevalence of drinking and driving was 21.0% (95%CI 19.9;23.2); among men it was 24.6% (95%CI 22.7;26.5) and among women, 10.1% (95%CI 7.9;12.7; p-value < 0.001). Among females, 4.8% of those who maintained this habit were motorcycle riders, 3.4% were car drivers and 1.9% were both car drivers and motorcycle riders; for males, 10.8% were both car drivers and motorcycle riders, 9.0% were car drivers and 4.8% were motorcycle riders (Figure 2).

The highest proportion of males were aged 35 to 59 years (49.3%), of Black race/skin color (Black and mixed-race: 73.8%), living without a partner (61.5%), with elementary education (39.8%) and *per capita* household income of up to ½ minimum wage (36.7%). The majority of men (73.7%) lived in urban areas and reported riding motorcycles (75.7%) and driving cars (65.6%). The highest prevalence of drinking and driving was observed in younger age groups, for those who reported living without a partner, those living in rural areas and those who reported riding a motorcycle (Table 1).

**Table 1:** Population characteristics and frequency of drinking and driving according to sociodemographic variables in the population, stratified by sex, National Health Survey, Northeastern Brazil, 2019

Variable	**Male (n = 4,984)**				Female (n = 1,355)		
	**Total** **(%)**	**Drinking and driving** **(%)**	**p-value** ^a^		**Total** **(%)**	**Drinking and driving** **(%)**	**p-value** ^a^
**Age range (years)**							
≥ 60	8.2	17.8	0.001		5.6	6.9	0.498
35-59	49.3	22.2			43.9	9.2	
18-34	42.5	28.5			50.5	11.1	
**Race/skin color**							
Black	73.8	24.4	0.738		66.9	11.4	0.093
White	26.2	25.1			33.1	7.6	
**Marital status**							
With a partner	38.5	22.3	0.045		29.5	7.5	0.112
Without a partner	61.5	26.0			70.5	11.1	
**Education level**							
Higher education	17.6	23.9	0.761		42.4	9.0	0.536
High school	37.4	23.6			39.2	11.2	
Elementary education	39.8	25.4			17.3	10.5	
Uneducated	5.2	26.9			1.2	2.4	
*Per capita* **household income (in minimum wages)**							
≤ ½	36.7	22.6	0.484		29.9	12.7	0.366
> ½ to 1	28.5	25.7			24.3	8.8	
> 1 to 2	19.5	25.6			18.6	7.1	
> 2	15.3	25.7			27.2	10.3	
**Place of residence**							
Urban area	73.7	22.5	< 0.001		81.6	9.0	0.112
Rural area	26.3	30.3			18.4	14.7	
**Drive a car**							
No	34.4	26.2	0.189		45.9	10.5	0.725
Yes	65.6	23.7			54.1	9.7	
**Ride a motorcycle**							
No	24.3	19.9	0.004		39.6	8.4	0.262
Yes	75.7	26.1			60.4	11.1	

a) Wald test.

Among females, 50.5% were aged 18 to 34 years, 66.9% self-identified as Black and 70.5% lived without a partner. A greater proportion had higher education (42.4%) and *per capita* household income of up to ½ minimum wage (29.9%). The majority of them (81.6%) lived in urban areas, rode motorcycles (60.4%) and drove cars (54.1%) (Table 1). Sociodemographic characteristics were not associated with drinking and driving among women.

Multiple regression analysis was performed only for males and showed a higher habit of drinking and driving in the age groups of 18 to 34 years (PR = 1.70 – 95%CI 1.29;2.24) and 35 to 59 years (PR = 1.34 – 95%CI 1.02;1.74), *per capita* household income greater than ½ minimum wage up to one minimum wage (PR = 1.33 – 95%CI 1.10-1.61), greater than one minimum wage up to two minimum wages (PR = 1.45 – 95%CI 1.17;1.79) and greater than two minimum wages (PR = 1.74 – 95%CI 1.33;2.28). Living in a rural area (PR = 1.48 – 95%CI 1.26;1.74) and riding a motorcycle (PR = 1.29 – 95%CI 1.05;1.58) were also factors positively associated with the habit of drinking and driving (Table 2).

**Table 2:** Crude and adjusted prevalence ratios, and 95% confidence interval for drinking and driving according to sociodemographic variables in the male population (n = 4,984), National Health Survey, Northeastern Brazil, 2019

Variable	Crude PR^a^ (95%CI ^b^)	p-value^c^	Adjusted PR^a^ (95%CI ^b^ )	p-value^c^
**Age range (years)**				
≥ 60	1.00		1.00	
35-59	1.25 (0.96;1.63)	0.101	1.34 (1.02;1.74)	0.032
18-34	1.60 (1.22;2.11)	0.001	1.70 (1.29;2.24)	< 0.001
**Race/skin color**				
Black	1.00			
White	1.03 (0.89;1.87)	0.738	**–**	**–**
**Marital status**				
With a partner	1.00			
Without a partner	1.16 (1.01;1.35)	0.045	**–**	**–**
**Education level**				
Higher education	1.00			
High school	0.98 (0.80;1.21)	0.887	**–**	**–**
Elementary education	1.06 (0.86;1.32)	0.575	**–**	**–**
Uneducated	1.12 (0.79;1.60)	0.517	**–**	**–**
*Per capita* **household income (in minimum wages)**				
≤ ½	1.00		1.00	
> ½ to 1	1.14 (0.94;1.37)	0.188	1.33 (1.10;1.61)	0.003
> 1 to 2	1.31 (0.92;1.40)	0.250	1.45 (1.17;1.79)	0.001
> 2	1.13 (0.90;1.42)	0.280	1.74 (1.33;2.28)	< 0.001
**Place of residence**				
Urban area	1.00		1.00	
Rural area	1.34 (1.49;1.57)	< 0.001	1.48 (1.26;1.74)	< 0.001
**Drive a car**				
No	1.00			
Yes	0.91 (0.78;1.05)	0.189	**–**	**–**
**Ride a motorcycle**				
No	1.00		1.00	
Yes	1.31 (1.09;1.57)	0.004	1.29 (1.05;1.58)	0.015

a) PR: prevalence ratio; b) 95%CI : 95% confidence interval; c) Wald test.

## DISCUSSION

The prevalence of drinking and driving was significantly higher among men when compared to women. Among males, younger age, higher income, living in rural areas and riding motorcycles were positively associated with drinking and driving. Among females, no significant associations were observed between sociodemographic characteristics and the outcome.

This study was based on self-reported variables, which may result in underestimation of the habit of drinking and driving. The PNS, in order to mitigate possible failures, implemented standardization procedures and provided training to interviewers responsible for data collection to ensure the quality of the data obtained.^12^

The prevalence of drinking and driving identified, above 20%, was higher than that found among Brazilians (5.4%).^6^ A study focusing on the macro-regions of Brazil regarding driving under the influence of alcohol, also found a high prevalence of drinking and driving in the Northeast region (29.4%), significantly higher than the prevalence in other regions of the country.^13^ The existing regional disparities, such as socioeconomic characteristics, access to education and cultural nuances, may justify the different prevalence of this risky behavior among the five macro-regions.^13^ Additionally, the existing weaknesses of laws and policies for monitoring alcohol consumption and life in traffic in Brazil, must be taken into consideration.^14,15^

Brazilian policies for alcohol consumption and enforcement have some gaps that could contribute to risk mitigation. Regarding pricing policies, the minimum taxation of undistilled alcoholic beverages favors their commercialization, as the sales prices of these products can be reduced and, combined with advertising and exposure strategies, induce early consumption among young people, including an increase in its frequency. There are significant legislative contradictions concerning the physical availability of alcohol, such as the lack of laws controlling retail sales of alcoholic beverages, in the essence of policies to control their commercialization that do not cover all types of alcoholic beverages, and consumption policies, favoring the acquisition and consumption of alcoholic beverages, especially by younger people.^14,15^ Despite the existence of laws and policies that monitor and regulate alcohol consumption, most of them, despite reinforcing the need for prevention and treatment interventions, do not provide the means for implementation.^14,15^

Although federal laws against driving under the influence of alcohol are up-to-date and more severe and punitive actions are in place,^14-16^ a high prevalence of drinking and driving is observed in Brazil, with the Northeast region standing out in this regard. The region is particularly known for its popular festivities and traditional events. Active participation in alcohol consumption during these social events, combined with the habit of driving home after these celebrations, may contribute to the high prevalence of drinking and driving in the region.^13,17,18^ Despite the traffic enforcement actions, proposed nationwide, which have led to a reduction in the habit of driving under the influence of alcohol,^19^ the Northeast region and its enforcement agencies still face challenges in this regard. Among these challenges are the low number of municipalities integrated into the National Traffic System, changes in driver behavior, such as blood alcohol level and reckless driving, low enforcement and the need for more traffic education actions.^20^ These factors reinforce the inequalities in the implementation of the Brazilian Traffic Code among the macro-regions of the country, especially the need to expand actions aimed at the Northeast region.

In this study, drinking and driving was more frequent among males. It is common knowledge that this population group not only shows a higher frequency of alcohol consumption but also greater involvement in traffic accidents.^4,15^ This association can be explained by men’s greater historical exposure to vehicle driving, combined with a cultural identification of virility in adopting aggressive behaviors behind the wheel.^10^ Furthermore, the influence of alcohol tends to increase impulsivity and risky behavior while driving, promoting reckless driving.^21^

Younger individuals from the Northeast region also showed higher prevalence of drinking and driving. Previous studies indicate an increase in the reporting of such behavior in this age group, followed by a decrease as age advances.^4,5,22^ The literature has already addressed the increase in alcohol consumption among young adults.^1,23^ The lack of monitoring alcohol consumption, combined with the alcohol industry’s marketing strategies targeting this specific population, justifies the adoption of this risky behavior in this group.^24^

Higher income was also positively associated with drinking and driving among males. A possible explanation for this would be a higher purchasing power, greater ease in acquiring motor vehicles and the habit of alcohol consumption; ^4^ additionally, there is greater participation in social events involving the presence of alcoholic beverages, their consumption and driving home afterward.^17,18^

Men who lived in rural areas had a higher prevalence of drinking and driving, whose motivations in rural areas may differ from those in urban areas. It is possible that long, sparsely traveled roads, with few transport alternatives, and a lack of enforcement explain the high prevalence of drinking and driving in rural contexts.^25^ In this same context, particularly in the Northeast region, the lack of education, associated with an intense urbanization process, plays a significant role in the prevalence of drinking and driving. Low level of education not only hinders the understanding of traffic rules, but also prevents the acquisition and regularization of the National Driver’s License, an essential legal requirement for driving vehicles.^9,26^ Another contributing factor in the rural world to the event studied: the advancement of tools used in agricultural activities and the increased use of agricultural machinery, causing many residents in these areas, even without formal training, to take on driving vehicles.^8^

A higher prevalence of drinking and driving was observed in men who reported riding motorcycles. Riding motorcycles after consuming alcoholic beverages is one of the leading causes of traffic accidents in Brazil, as indicated by the Ministry of Health in 2023.^26^ Between 2019 and 2021, 13,415 deaths from this cause were recorded in the Northeast region,^27^ with a large population immersed in contexts of socioeconomic vulnerability, marked by high unemployment rates. For many individuals, the motorcycle has become an alternative for income generation,^10,28^ especially in informal work. Informal workers, and a paradigmatic example of this condition, app delivery riders, depend on motorcycles for their daily activities, mainly due to the agility, mobility and low operating cost of these vehicles.^29^ The delivery service is driven by the increase in online services and fast delivery. However, the expansion of these services brings challenges, such as social protection deficiencies, lack of benefits and, consequently, concrete harm to these workers’ lives.^29^

Moto-taxi riders and motorcycle couriers often work in precarious conditions, paid based on production. Working hours exceeding ten hours a day, speeding and the use of cell phones while riding significantly increase the chances of accidents and deaths among this population.^30^ Under these working conditions, socioeconomic factors such as low level of education and low pay, more pronounced in the Northeast region, can lead to risky behaviors, including alcohol consumption.^10,30^

Northeastern Brazil stands out for presenting a worrying scenario regarding accidents involving motorcyclists. The roots of this problem are complex, encompassing educational and macroeconomic determinants that directly influence the adoption of risky behaviors in traffic. This finding reiterates the inherent inequalities in the region.^9^

Taking into consideration the social and economic implications of the health conditions attributed to the alcohol use and driving vehicles,^9,25^ it is suggested that intersectoral actions be established in order to reduce this risky behavior, expanding investments in health and education, aimed to target all population groups. More effective enforcement measures and health and traffic education actions must be reinforced to prevent this practice, especially among young people.

In conclusion, the results of this study strengthen the need for the implementation of more effective public policies focused on young people. It is worth highlighting the crucial importance of increasing traffic enforcement by the authorities. These are imperative measures to mitigate the negative impacts of accidents, especially those involving motorcyclists, and contribute to the construction of a safer and more equitable road environment in the Northeast region of Brazil.
