# Supplementary material for: Driving after alcohol consumption among residents of Northeastern Brazil: National Health Survey 2019
Source: Epidemiol Serv Saude. 2024 Oct 14;33:e2024455. doi: 10.1590/S2237-96222024v33e2024455.en (PMC11473066; doi:10.1590/S2237-96222024v33e2024455.en)
Supplement: Translation

# Translation: Condução de veículos após o consumo de bebidas alcoólicas em residentes do Nordeste do Brasil: Pesquisa Nacional de Saúde 2019

**DOI:** 10.1590/S2237-96222024v33e2024455.pt

**Authors:** Renata da Silva Gomes, Amanda Cristina de Souza Andrade, Vanessa Moraes Bezerra

**Affiliations:** 1 Universidade Federal da Bahia, Programa de Pós-Graduação em Saúde Coletiva, Vitória da Conquista, BA, Brasil; 2 Universidade Federal de Mato Grosso, Instituto de Saúde Coletiva, Cuiabá, MT, Brasil

**Keywords:** Acidentes de Trânsito, Estudos Transversais, Consumo de Bebidas Alcoólicas, Comportamentos de Risco à Saúde, Inquéritos Epidemiológicos

## Abstract

**Objetivo:**

Estimar a prevalência e analisar fatores associados ao hábito de beber e dirigir no Nordeste do Brasil.

**Métodos:**

Estudo transversal, com participantes da Pesquisa Nacional de Saúde 2019, aos 18 anos ou mais de idade; analisou-se a associação entre variáveis sociodemográficas e o desfecho, estratificada segundo sexo, mediante regressão de Poisson.

**Resultados:**

A prevalência de “beber e dirigir” de 21,0% (IC_95_% 19,9;23,2), 24,6% (IC_95_% 22,7;26,5) no sexo masculino e de 10,1% (IC_95_% 7,9;12,7) no feminino (p-valor < 0,001); nos homens, faixas etárias mais jovens (RP = 1,70 – IC_95_% 1,29;2,24), maior renda domiciliar (RP = 1,74 – IC_95_% 1,33;2,28), residência rural (RP = 1,48 – IC_95_% 1,26;1,74) e condução de motocicleta (RP = 1,29 – IC_95_% 1,05;1,58) associaram-se ao evento, enquanto nenhuma associação foi observada nas mulheres.

**Conclusão:**

A prevalência de “beber e dirigir” alta no Nordeste, comparada à do Brasil, principalmente na população masculina; fazem-se necessárias medidas preventivas nesse público, e intensificação da fiscalização.

## Contribuições do estudo

**Principais resultados:**

Prevalência do hábito de “beber e dirigir” elevada, significativamente maior no sexo masculino frente ao feminino; apenas no sexo masculino foram observadas associações positivas de variáveis sociodemográficas com o evento.

**Implicações para os serviços:**

Ações intersetoriais, a envolver serviços de saúde e órgãos de regulação, são essenciais para reduzir o hábito de beber e dirigir, especialmente no sexo masculino, além de se promover um ambiente viário mais seguro no Nordeste.

**Perspectivas:**

A necessidade do fortalecimento das medidas regulatórias de fiscalização de álcool e direção, especialmente para áreas rurais e aos condutores de motocicletas. Além de ações de educação no trânsito e campanhas de conscientização.

## INTRODUÇÃO

O álcool é uma droga lícita, amplamente utilizada por homens e mulheres e difundida em diversas culturas, associada a festividades, cerimônias religiosas e celebrações. Apesar de favorecer a socialização e integração entre os indivíduos, o consumo de bebidas alcoólicas em grandes quantidades pode acarretar consequências prejudiciais para a saúde do indivíduo e para a sociedade.^1,2^

A prática de dirigir após ingerir bebidas alcoólicas é uma das principais causas de acidentes de trânsito no mundo.^2^ Em países de renda elevada, aproximadamente 20% dos motoristas envolvidos em acidentes fatais apresentaram álcool no sangue, ao passo que, em alguns países de baixa e média renda, esse percentual foi três vezes maior.^2^

Mesmo em quantidades reduzidas, o álcool induz modificações psicomotoras, como deterioração das funções visuais e motoras, além de reduzir a capacidade de discernimento, implicando riscos substanciais para o condutor de veículo, seus passageiros e pedestres.^3,4^ Quanto aos fatores de risco associados ao hábito de beber e dirigir, destacam-se o sexo masculino, faixas etárias mais jovens e maior renda.^4,5^

No Brasil, em 2021, os acidentes de trânsito destacaram-se como a principal razão para internações relacionadas com o consumo de bebidas alcoólicas – 22,6% do total – e a segunda causa mais prevalente de mortalidade associada ao uso dessa substância – 15,8% do total.^6^ As maiores taxas de mortalidade por acidentes de trânsito no país corresponderam às macrorregiões Norte, Nordeste e Centro-Oeste, afetando principalmente homens, indivíduos mais jovens e aqueles com maior renda. 

Observou-se queda de 43% nas taxas de mortalidade por acidentes de trânsito no Brasil, entre 1990 e 2019.^6^ Apesar desse avanço importante, a meta de reduzir em 50% as mortes por acidente de trânsito no Brasil, até 2030, pode não ser alcançada.^7^

Além da alta mortalidade no trânsito, especialmente de motociclistas, o Nordeste brasileiro enfrenta profundas desigualdades em sua população, sobretudo relacionadas às condições sociais e econômicas. A região é conhecida por contar com menor índice de escolaridade e renda, e piores condições de saúde. A coexistência desses fatores não só está associada ao consumo elevado de álcool como também representa riscos para o hábito de beber e dirigir.^8,9^

Elevadas morbimortalidades por acidentes de trânsito em decorrência do consumo de bebidas alcoólicas, e suas consequências, produzem efeitos para além dos gastos hospitalares e dos custos no cuidado à saúde.^4,10^ Trata-se de uma questão e um desafio à saúde pública, em todo o mundo, e o Nordeste do Brasil não está imune a isso.

Muito embora seja evidente a relevância e os potenciais impactos negativos na saúde do indivíduo e na sociedade, verifica-se uma lacuna notável na literatura científica que investiga esse fenômeno no Nordeste. A escassez de estudos sobre o consumo de bebidas alcoólicas na região limita a compreensão dos padrões de consumo, dos fatores de risco envolvidos e das consequências para a saúde pública. 

O presente estudo teve por objetivo avaliar a prevalência e fatores sociodemográficos associados ao hábito de beber e dirigir entre residentes do Nordeste do Brasil, estratificados por sexo.

## MÉTODOS

Trata-se de estudo transversal, realizado com dados da Pesquisa Nacional de Saúde (PNS), em sua edição de 2019,^11^ sobre adultos residentes na região Nordeste do Brasil.

A coleta de dados da PNS 2019 ocorreu entre agosto de 2019 e março de 2020. A Pesquisa adotou um delineamento amostral, por conglomerado, em três estágios: os setores censitários; os domicílios; e um indivíduo com 15 anos ou mais de idade, residente no domicílio, escolhido de forma aleatória.^11,12^

Em números totais, a PNS 2019 contou com 91.683 respondentes na idade de 18 anos ou mais, e destes, 31.800 eram moradores do Nordeste.^12^ Na amostra deste estudo, foram considerados aqueles com 18 anos ou mais que responderam às perguntas sobre o consumo de bebidas alcoólicas, e os que conduziam carros e/ou motos, totalizando 6.339 indivíduos (Figura 1).

**Figura 1:** Processo de identificação de indivíduos de 18 anos ou mais que relataram o hábito de beber e dirigir, Pesquisa Nacional de Saúde, Nordeste do Brasil, 2019

A variável dependente do estudo foi “beber e dirigir” enquanto hábito. Este indicador foi construído a partir da seguinte pergunta da PNS: *Em algum destes dias em que consumiu bebida alcoólica, o(a) Sr.(a) dirigiu logo depois de beber?*

A pergunta contava com duas opções de resposta, “sim” e “não”, propostas unicamente aos indivíduos que informaram consumir bebida alcoólica e àqueles que dirigiam automóvel ou motocicleta. Os condutores foram identificados pela pesquisa a partir das seguintes perguntas: *Atualmente, o(a) Sr.(a) dirige automóvel (inclusive táxi, aplicativos de transportes e similares)? Atualmente, o(a) Sr.(a) dirige motocicleta?*

A análise foi estratificada pela variável “sexo” (masculino; feminino). O hábito de beber e dirigir foi analisado segundo as características sociodemográficas a seguir.

faixa etária (em anos: 18 a 34; 35 a 59; 60 ou mais);
raça/cor da pele [classificada em duas categorias, brancos e negros (estes, pretos e pardos); foram excluídos da análise os que se autodeclararam de raça/cor da pele amarela e os indígenas];
situação conjugal [sem companheiro(a); com companheiro(a)];
nível de escolaridade [sem instrução; ensino fundamental (incompleto/completo); ensino médio (incompleto/completo); ensino superior [incompleto/completo]);
renda domiciliar *per capita* (em salários mínimos: ≤ ½; ½ até 1; >1 até 2; > 2);
local de residência (área urbana; área rural);
dirige automóvel (sim; não); e
dirige motocicleta (sim; não).

Para a análise de dados, foi utilizado o *software* Stata/SE em sua versão 15 e o módulo Survey, que considera o desenho complexo da amostra e o peso amostral. A prevalência do evento foi calculada segundo as características sociodemográficas. Foram realizadas análises bivariadas e múltiplas, com estimativas de razões de prevalências (RPs) e respectivos intervalos de confiança de 95% (IC_95_%), mediante regressão de Poisson com variância robusta. Para a análise múltipla, adotou-se o método de seleção *backward*, sendo incluídas todas as variáveis sociodemográficas; uma variável por vez foi eliminada do modelo. Foram mantidas no modelo final apenas as variáveis que apresentaram p-valor ≤ 0,05. 

O projeto da PNS 2019 foi aprovado pela Comissão Nacional de Ética em Pesquisa do Conselho Nacional de Saúde (Conep/CNS): Parecer nº 3.529.376.

## RESULTADOS

Do total de entrevistados (n = 6.339 indivíduos), 79,0% eram do sexo masculino e 21,0% do sexo feminino; a prevalência do hábito de beber e dirigir foi de 21,0% (IC_95_% 19,9;23,2) para esse total, do qual 24,6% (IC_95_% 22,7;26,5) eram homens e 10,1% (IC_95_% 7,9;12,7) mulheres (p-valor < 0,001). Ao descrever o hábito de beber e dirigir segundo “sexo” e “tipo de veículo”, para o sexo feminino, 4,8% eram condutoras de motocicleta, 3,4% de carro e 1,9% de carro e motocicleta; para o sexo masculino, 10,8% eram condutores de carro e motocicleta, 9,0% de carro e 4,8% de motocicleta (Figura 2).

**Figura 2:** Prevalência do hábito de beber e dirigir, segundo o tipo de veículo, na população com 18 anos ou mais de idade, estratificada por sexo, Pesquisa Nacional de Saúde, Nordeste do Brasil, 2019

No sexo masculino, as maiores proporções corresponderam à idade de 35 a 59 anos (49,3%), raça/cor da pele negra (pretos e pardos: 73,8%), viver sem companheira(o) (61,5%), ensino fundamental incompleto/completo (39,8%) e renda domiciliar *per capita* de até ½ salário mínimo (36,7%). A maioria dos homens (73,7%) residiam na área urbana, afirmaram dirigir motocicleta (75,7%) e automóvel (65,6%). As maiores prevalências do hábito de beber e dirigir foram observadas nas faixas etárias mais jovens, para os que relataram viver sem companheira(o), nos que viviam em área rural e naqueles que afirmaram dirigir motocicleta (Tabela 1).

**Tabela 1:** Características da população e frequência do hábito de beber e dirigir segundo variáveis sociodemográficas na população, estratificada por sexo, Pesquisa Nacional de Saúde, Nordeste do Brasil, 2019

Variável	Masculino (n = 4.984)				Feminino (n = 1.355)		
	**Total** **(%)**	**Beber e dirigir** **(%)**	**p-valor** ^c^		**Total** **(%)**	**Beber e dirigir** **(%)**	**p-valor** ^c^
**Faixa etária (anos)**							
≥ 60	8,2	17,8	0,001		5,6	6,9	0,498
35-59	49,3	22,2			43,9	9,2	
18-34	42,5	28,5			50,5	11,1	
**Raça/cor da pele**							
Negros	73,8	24,4	0,738		66,9	11,4	0,093
Brancos	26,2	25,1			33,1	7,6	
**Situação conjugal**							
Com companheiro (a)	38,5	22,3	0,045		29,5	7,5	0,112
Sem companheiro (a)	61,5	26,0			70,5	11,1	
**Nível de escolaridade**							
Superior (incompleto/completo)	17,6	23,9	0,761		42,4	9,0	0,536
Médio (incompleto/completo)	37,4	23,6			39,2	11,2	
Fundamental (incompleto/completo)	39,8	25,4			17,3	10,5	
Sem instrução	5,2	26,9			1,2	2,4	
**Renda domiciliar** *per capita* **(em salários mínimos)**							
≤ ½	36,7	22,6	0,484		29,9	12,7	0,366
> ½ até 1	28,5	25,7			24,3	8,8	
> 1 até 2	19,5	25,6			18,6	7,1	
> 2	15,3	25,7			27,2	10,3	
**Local de residência**							
Área urbana	73,7	22,5	< 0,001		81,6	9,0	0,112
Área rural	26,3	30,3			18,4	14,7	
**Dirige automóvel**							
Não	34,4	26,2	0,189		45,9	10,5	0,725
Sim	65,6	23,7			54,1	9,7	
**Dirige motocicleta**							
Não	24,3	19,9	0,004		39,6	8,4	0,262
Sim	75,7	26,1			60,4	11,1	

a) Teste de Wald.

No sexo feminino, ligeira maioria (50,5%) tinha entre 18 e 34 anos de idade, 66,9% foram classificadas como negras e 70,5% viviam sem companheiro(a). Sobre o nível de escolaridade, em maior proporção, encontrou-se o ensino superior incompleto/completo (42,4%) e a renda domiciliar *per capita* até ½ salário mínimo (29,9%). Ainda sobre o sexo feminino, a localização da residência e a condução de veículos, 81,6% viviam na área urbana, 54,1% dirigiam automóvel e 60,4% motocicleta. Características sociodemográficas não foram associadas ao hábito de beber e dirigir, entre as mulheres (Tabela 1).

A análise de regressão múltipla foi realizada apenas para o sexo masculino. Ela demonstrou associação ao hábito de beber e dirigir nas faixas etárias de 18 a 34 anos (RP = 1,70 – IC_95_% 1,29;2,24) e 35 a 59 anos (RP = 1,34 – IC_95_% 1,02;1,74), na renda domiciliar *per capita* maior que ½ até um salário mínimo (RP = 1,33 – IC_95_% 1,10-1,61), maior que um salário até dois salários mínimos (RP = 1,45 – IC_95_% 1,17;1,79) e maior que dois salários mínimos (RP = 1,74 – IC_95_% 1,33;2,28). A associação de “idade” e “renda” com o evento apresentou uma relação dose-resposta. Residir na zona rural (RP = 1,48 – IC_95_% 1,26;1,74) e dirigir motocicleta (RP = 1,29 – IC_95_% 1,05;1,58) também foram fatores positivamente associados à variável dependente “beber e dirigir” (Tabela 2).

**Tabela 2:** Razão de prevalências, bruta e ajustada, e intervalo de confiança de 95%, para hábito de beber e dirigir segundo variáveis sociodemográficas na população do sexo masculino (n = 4.984), Pesquisa Nacional de Saúde, Nordeste do Brasil, 2019

Variável	RP^a^ bruta (IC_95_ **%** ^b^)	p-valor^c^	RP^a^ ajustada (IC_95_ **%** ^b^)	p-valor^c^
**Faixa etária (anos)**				
≥ 60	1,00		1,00	
35-59	1,25 (0,96;1,63)	0,101	1,34 (1,02;1,74)	0,032
18-34	1,60 (1,22;2,11)	0,001	1,70 (1,29;2,24)	< 0,001
**Raça/cor da pele**				
Negros	1,00			
Brancos	1,03 (0,89;1,87)	0,738	**–**	**–**
**Situação conjugal**				
Com companheiro (a)	1,00			
Sem companheiro (a)	1,16 (1,01;1,35)	0,045	**–**	**–**
**Nível de escolaridade**				
Superior (incompleto/completo)	1,00			
Médio (incompleto/completo)	0,98 (0,80;1,21)	0,887	**–**	**–**
Fundamental (incompleto/completo)	1,06 (0,86;1,32)	0,575	**–**	**–**
Sem instrução (incompleto/completo)	1,12 (0,79;1,60)	0,517	**–**	**–**
**Renda domiciliar** *per capita* **(em salários mínimos)**				
≤ ½	1,00		1,00	
> ½ até 1	1,14 (0,94;1,37)	0,188	1,33 (1,10;1,61)	0,003
> 1 até 2	1,31 (0,92;1,40)	0,250	1,45 (1,17;1,79)	0,001
> 2	1,13 (0,90;1,42)	0,280	1,74 (1,33;2,28)	< 0,001
**Local de residência**				
Área urbana	1,00		1,00	
Área rural	1,34 (1,49;1,57)	< 0,001	1,48 (1,26;1,74)	< 0,001
**Dirige automóvel**				
Não	1,00			
Sim	0,91 (0,78;1,05)	0,189	**–**	**–**
**Dirige motocicleta**				
Não	1,00		1,00	
Sim	1,31 (1,09;1,57)	0,004	1,29 (1,05;1,58)	0,015

a) RP: Razão de prevalências; b) IC_95_%: Intervalo de confiança de 95%; c) Teste de Wald.

## DISCUSSÃO

Neste estudo, a prevalência do hábito de beber e dirigir foi significativamente maior entre os homens, frente às mulheres. No sexo masculino, foram observadas associações de faixas etárias mais jovens, maiores níveis de renda domiciliar *per capita*, residir em área rural e dirigir motocicletas com o hábito de beber e dirigir.

No que concerne às limitações da presente investigação, destaca-se o fato de os indicadores serem autorreferidos, com possibilidade de subestimação do hábito de beber e dirigir. Entretanto, cumpre destacar que a PNS, para mitigar possíveis falhas, implementou procedimentos de padronização e ofereceu treinamento aos entrevistadores responsáveis pela coleta das informações, com vistas a assegurar a qualidade dos dados obtidos.^12^

A prevalência do hábito de beber e dirigir identificada foi substancialmente superior, atingindo 22,1%, enquanto a prevalência encontrada entre os brasileiros foi de 5,4%.^6^ Um estudo focado nas grandes regiões brasileiras, com o tema da direção sob efeito do álcool, também observou prevalência elevada do hábito de beber e dirigir no Nordeste (29,4%), notadamente superior às prevalências das demais regiões do país.^13^ As disparidades regionais existentes, como características socioeconômicas, acesso a educação e nuances culturais, podem justificar as distintas prevalências desse comportamento de risco entre as cinco macrorregiões.^13^ Também é importante considerar as fragilidades existentes nas legislações e nas políticas de fiscalização do consumo de álcool e da vida no trânsito, no Brasil.^14,15^

Em relação às políticas brasileiras de fiscalização e consumo de álcool, é possível apontar algumas lacunas. Nas políticas de preços, principalmente, a tributação mínima de bebidas alcoólicas não destiladas favorece sua comercialização, na medida em que os preços de venda desses produtos possam ser reduzidos e, somados ao *marketing* de publicidade e estratégias de exposição, induzam seu consumo precoce entre os jovens, inclusive o aumento de sua frequência. Outrossim, há importantes contradições legislativas quanto à disponibilidade física do álcool, como a inexistência de leis de controle da venda de bebidas no varejo, no conteúdo das políticas para controlar sua comercialização – que não abrangem todos os tipos de bebidas alcoólicas –, e nas políticas de consumo, favorecendo a aquisição e o consumo das bebidas alcoólicas, principalmente pelos mais jovens.^14,15^ Apesar da existência de leis e políticas que fiscalizam e regulam o consumo de bebidas alcoólicas, grande parte delas, apesar de reforçarem a necessidade de intervenções de prevenção e tratamento, não dispõem dos meios para serem executadas.^14,15^

Embora as leis federais contra a condução de veículos sob o efeito do álcool estejam atualizadas e com ações mais severas e punitivas,^14-16^ são observadas prevalências elevadas do hábito de beber e dirigir no Brasil, destacando-se o Nordeste nessa condição. A região é conhecida especialmente por suas festas populares e eventos tradicionais. A participação ativa no consumo de álcool durante esses eventos sociais, aliada à prática de dirigir na volta para casa após essas celebrações, pode contribuir com a prevalência elevada do hábito de beber e dirigir na região.^13,17,18^ A despeito das ações de fiscalização de trânsito, propostas em âmbito nacional, terem levado à redução da prática de conduzir veículos sob efeito do álcool,^19^ o Nordeste e seus órgãos de fiscalização, todavia, enfrentam algumas dificuldades nesse sentido. Entre essas dificuldades, está o baixo índice de municípios integrados ao Sistema Nacional de Trânsito (SNT), as mudanças de comportamento dos condutores, como a alcoolemia e as imprudências no trânsito, a baixa fiscalização e a necessidade de mais ações de educação no trânsito.^20^ Estes fatores reforçam as desigualdades na implantação do Código de Trânsito Brasileiro (CTB) entre as grandes regiões do país, especialmente a necessidade de ampliação das ações dirigidas ao Nordeste. 

No presente trabalho, o hábito de beber e dirigir foi mais prevalente entre indivíduos do sexo masculino. Sabe-se que esse grupo populacional apresenta não apenas uma maior frequência de consumo de bebidas alcoólicas, mas também maior participação em acidentes de trânsito.^4,15^ Esta associação pode ser explicada pela maior exposição histórica dos homens à direção de veículos, aliada a uma identificação cultural de virilidade na adoção de comportamentos agressivos ao volante.^10^ Além do que, a influência do álcool tende a aumentar a impulsividade e o comportamento arriscado na condução de veículos, fomentando ações imprudentes no trânsito.^21^

Nordestinos mais jovens também apresentaram maiores prevalências do hábito de beber e dirigir. Estudos anteriores evidenciam um aumento no relato de tal comportamento nessa faixa etária, seguido de uma diminuição à medida que a idade avança.^4,5,22^ A literatura já aborda o aumento do consumo de bebidas alcoólicas entre adultos jovens.^1,23^ A falta de fiscalização no consumo de álcool, somada ao estímulo da indústria do álcool com estratégias de *marketing* desenvolvidas para esse público específico, justifica a adoção desse comportamento de risco nesse grupo.^24^

Maiores níveis de renda domiciliar *per capita* também foram positivamente associados ao hábito de beber e dirigir entre indivíduos do sexo masculino. Uma possível explicação para isso estaria no maior poder aquisitivo, mais facilidades para a aquisição de veículos automotores e o consumo habitual de bebidas alcoólicas;^4^ a isto, acrescenta-se a maior participação em eventos de socialização com a presença de bebidas alcoólicas, seu consumo e a condução de veículo na volta para casa.^17,18^

Em relação ao local de residência, homens que viviam em área rural apresentaram maior prevalência do hábito de beber e dirigir, cujas motivações – em áreas rurais – podem ser diferentes nas áreas urbanas. É possível que estradas longas e pouco movimentadas, com poucas alternativas de transporte e ausência de fiscalização, expliquem a elevada prevalência de beber e dirigir no contexto rural.^25^ Neste mesmo contexto, particularmente no Nordeste, a carência educacional, associada a um intenso processo de urbanização, desempenha papel significativo na prevalência do hábito de beber e dirigir. A baixa escolaridade não apenas dificulta a compreensão das normas de trânsito – ela impede a aquisição e regularização da Carteira Nacional de Habilitação (CNH), requisito essencial, por lei, para a condução de veículos.^9,26^ E mais um fator contributivo do mundo rural para o evento estudado: a evolução das ferramentas utilizadas nas atividades agrárias e o aumento do uso de máquinas agrícolas fazem com que muitos residentes nessas áreas, mesmo sem capacitação formal, assumam a condução de veículos.^8^

Uma maior prevalência do hábito de beber e dirigir foi observada em homens que afirmaram dirigir motocicleta. A condução de motocicletas, após o consumo de bebidas alcoólicas, é uma das principais causas de acidentes de trânsito no Brasil, como indicou o Ministério da Saúde em 2023.^26^ No período de 2019 a 2021, foram registrados 13.415 óbitos por essa causa no Nordeste,^27^ região com grande contingente populacional imerso em contextos de vulnerabilidade socioeconômica, marcado por taxas elevadas de desemprego. Para muitos, a motocicleta tornou-se uma alternativa para obtenção de renda,^10,28^ sobretudo do trabalho informal. Trabalhadores na informalidade e, especialmente, um exemplo paradigmático dessa condição, os entregadores de aplicativos dependem das motocicletas para realizar suas atividades diárias, uma escolha principalmente relacionada à agilidade, mobilidade e baixo custo operacional desses veículos.^29^ O serviço de entregas é impulsionado pelo aumento do comércio de serviços *online* e entrega rápida. Contudo, a expansão desses serviços traz desafios, como desproteção social, ausência de benefícios e, por conseguinte, prejuízos concretos à vida desses trabalhadores.^29^

Mototaxistas e *motoboys* costumam desempenhar suas funções em condições precárias, remuneradas por produção. Jornadas de trabalho que ultrapassam dez horas diárias, excesso de velocidade e uso de dispositivos celulares durante a pilotagem ampliam substancialmente as chances de acidentes e óbitos nessa população.^30^ Sob tais condições de trabalho, fatores socioeconômicos como baixa escolaridade e baixa remuneração, mais acentuados na região Nordeste, podem levar a comportamentos de risco, entre os quais o consumo de bebidas alcoólicas.^10,30^

O Nordeste brasileiro se destaca por apresentar um cenário preocupante no que concerne aos acidentes envolvendo motociclistas. As raízes desse problema são complexas, abarcam determinantes educacionais e macroeconômicos, de influência direta na adoção de comportamentos de risco no trânsito. Essa constatação reitera as desigualdades inerentes à região.^9^

Considerando-se as implicações sociais e econômicas dos agravos atribuídos ao uso de bebidas alcoólicas e condução de veículos,^9,25^ sugere-se (i) o estabelecimento de ações intersetoriais envolvendo órgãos da Saúde, Educação e Regulação, visando cessar esse comportamento de risco, (ii) a expansão de investimentos em saúde e educação, para estes alcançarem, de maneira equitativa, todos os grupos populacionais, e (iii) a tomada de medidas de fiscalização mais efetivas no cumprimento das leis e ações de educação em saúde e no trânsito, com o objetivo de prevenir essa prática, principalmente entre os jovens. 

Em conclusão, os resultados do presente estudo fortalecem a necessidade da implementação de políticas públicas mais efetivas, focadas nos jovens. Ressaltamos a importância crucial do aumento das fiscalizações, por parte das autoridades de trânsito. São medidas imperativas no sentido de mitigar os impactos negativos dos acidentes, especialmente quando envolvem motociclistas, e contribuir para a construção de um ambiente viário mais seguro e equitativo no Nordeste do Brasil.
